# Subinguinal Orchidectomy for Testicular Cancer: Innovation or Unnecessary Advancement

**DOI:** 10.7759/cureus.86062

**Published:** 2025-06-15

**Authors:** Dayan Jacob, Pragnitha Chitteti, Mohamed Mubarak, Mehwash Nadeem

**Affiliations:** 1 Urology, James Cook University Hospital, Middlesbrough, GBR

**Keywords:** high orchidectomy, orchidectomy, testicular neoplasms, urology oncology, urology surgery

## Abstract

Subinguinal orchidectomy is a variation of the traditional high inguinal approach for testicular cancer, differing in the level of spermatic cord excision. While the subinguinal approach preserves the inguinal canal and ilioinguinal nerve, concerns remain about the residual disease in the proximal cord and its impact on oncological outcomes. This review evaluates oncological outcomes and complications of subinguinal orchidectomy. A search was conducted across five databases (PubMed, Scopus, Google Scholar, Cochrane Library, and Embase). Studies reporting inguinal versus subinguinal orchidectomies, tumor grade, oncological outcomes, complications, and follow-up were included. Descriptive statistics were performed using Microsoft Excel (Microsoft Corporation, Redmond, WA). Of the 25 studies screened, two were eligible for review, including data from 264 patients (2000-2024). Subinguinal orchidectomy was done in 54.7% (n=144) of cases. Unsatisfactory oncological control was observed in 12.5% (n=18) of subinguinal cases, with 78% (n=14) due to cancer relapse, 16.5% (n=3) due to spermatic cord invasion (SCI), and 5.5% (n=1) due to positive margins. One study comparing subinguinal and high inguinal approaches found no differences in oncological outcomes for stage 1 and stage 2-4 cancers (p=0.91 and p=0.78, respectively). One study reported that 9.5% of patients who underwent subinguinal orchidectomy (n=4) developed seromas postoperatively. Current evidence, though limited, suggests no significant differences in oncological outcomes between subinguinal and high-inguinal orchidectomies. While retrospective studies support this, prospective trials are required to better evaluate the oncological risk-benefit ratio of subinguinal orchidectomy.

## Introduction and background

Testicular cancer is the most common cancer among young men aged 15 to 34, accounting for less than 1% of all male tumors but approximately 20% of cancers in this age group in the UK [1,2]. The UK also has one of the highest age-standardized incidence rates for testicular cancer globally at 6.3 per 100,000 men, with similar increasing trends observed across developed countries [1-3].

The current treatment of choice in men with suspected primary testicular cancer is a radical inguinal orchiectomy, as it provides both oncological control and an adequate histopathological specimen [4]. Following staging completion, patients are placed on surveillance, receive chemoradiotherapy, or proceed to retroperitoneal lymph node dissection (RPLND) [5]. The introduction of platinum-based chemotherapy has significantly improved survival rates for testicular cancer from 46% pre-1978 to almost 95% in the 1990s [3,6].

From a surgical perspective, the classic high-cord radical inguinal orchiectomy is a well-established and widely adopted technique that involves high ligation of the spermatic cord at the internal ring and the removal of the ipsilateral spermatic cord, testis, and surrounding tunica vaginalis as a single unit [7].

Although the procedure is well-tolerated, it is associated with a number of postoperative complications. Examples include postoperative infections, bleeding, risk of inguinal hernias, and nerve injury [8]. Of note, damage or compression of the ilioinguinal nerve can lead to paresthesia and significant chronic postoperative pain, which often responds poorly to analgesics.

The “low-cord” or subinguinal approach to orchidectomy involves the identification, isolation, and ligation of the spermatic cord at the level of the superficial inguinal ring [5]. This technique could theoretically have a lower risk of complications, especially those involving the ilioinguinal nerve. The evidence to support this assumption is that subinguinal varicocelectomies have been shown to have a lower incidence of postoperative pain when compared to the inguinal approach [9,10]. However, with low-cord orchiectomies being performed for testicular cancer, there is a theoretical risk of incomplete cancer resection with tumor remnants still being left in the proximal spermatic cord.

Our study aims to review whether low-cord orchidectomies have poor oncological control versus the high-cord approach and whether there are differences in complication rates between the two approaches.

## Review

Methods

Search Strategy

This systematic review was designed and conducted in accordance with the PRISMA (Preferred Reporting Items for Systematic Reviews and Meta-Analyses) guidelines [11] and was prospectively registered with PROSPERO (registration number: CRD42024593767). A thorough search of multiple electronic databases was conducted to identify relevant studies published from 2000 to 2024. The databases searched included PubMed, Google Scholar, EMBASE, Cochrane Library, Scopus, and Web of Science. Eligible studies involved adult patients (aged 18 years or older) who had undergone a subinguinal orchidectomy as a treatment for testicular cancer, with subsequent follow-up including histopathological outcomes and clinical surveillance. We excluded certain study types, such as meta-analyses, systematic reviews, editorials, and expert opinions, to focus on original research. Case reports were excluded as they lacked a comparison between the two approaches. The primary outcome was oncological control after surgery, measured by negative histopathological margins or absence of recurrence at follow-up. The secondary outcome was the rate of adverse events. No language restrictions were imposed on the publications. The search strategy was informed by the PICO question, and the final search string was (Inguinal AND (Sub Inguinal OR Sub-inguinal OR subinguinal) AND (Orchiectomy OR orchidectomy)) AND (Cancer OR oncology OR oncological).

Data Selection and Screening Process

Once the initial search was completed, two independent reviewers (DJ and PC) screened the articles based on titles and abstracts. The studies deemed relevant were imported into Rayyan.ai [12], a tool designed to organize and eliminate duplicates automatically. Full-text articles were retrieved for those studies that passed the initial screening. Both reviewers (DJ and PC) then evaluated the eligibility of these studies based on their design, population, and reported outcomes. Any disagreements regarding study inclusion were resolved through discussion, and where necessary, a third reviewer (MM) was consulted to reach a consensus.

*Data Extraction and Analysis * 

Data extraction for the eligible studies was performed using Microsoft Excel (Microsoft Corporation, Redmond, WA). The extracted data included relevant outcomes such as oncological control and adverse events. Quantitative analysis was conducted using Microsoft Excel for descriptive statistics.

Results

**Figure 1:**
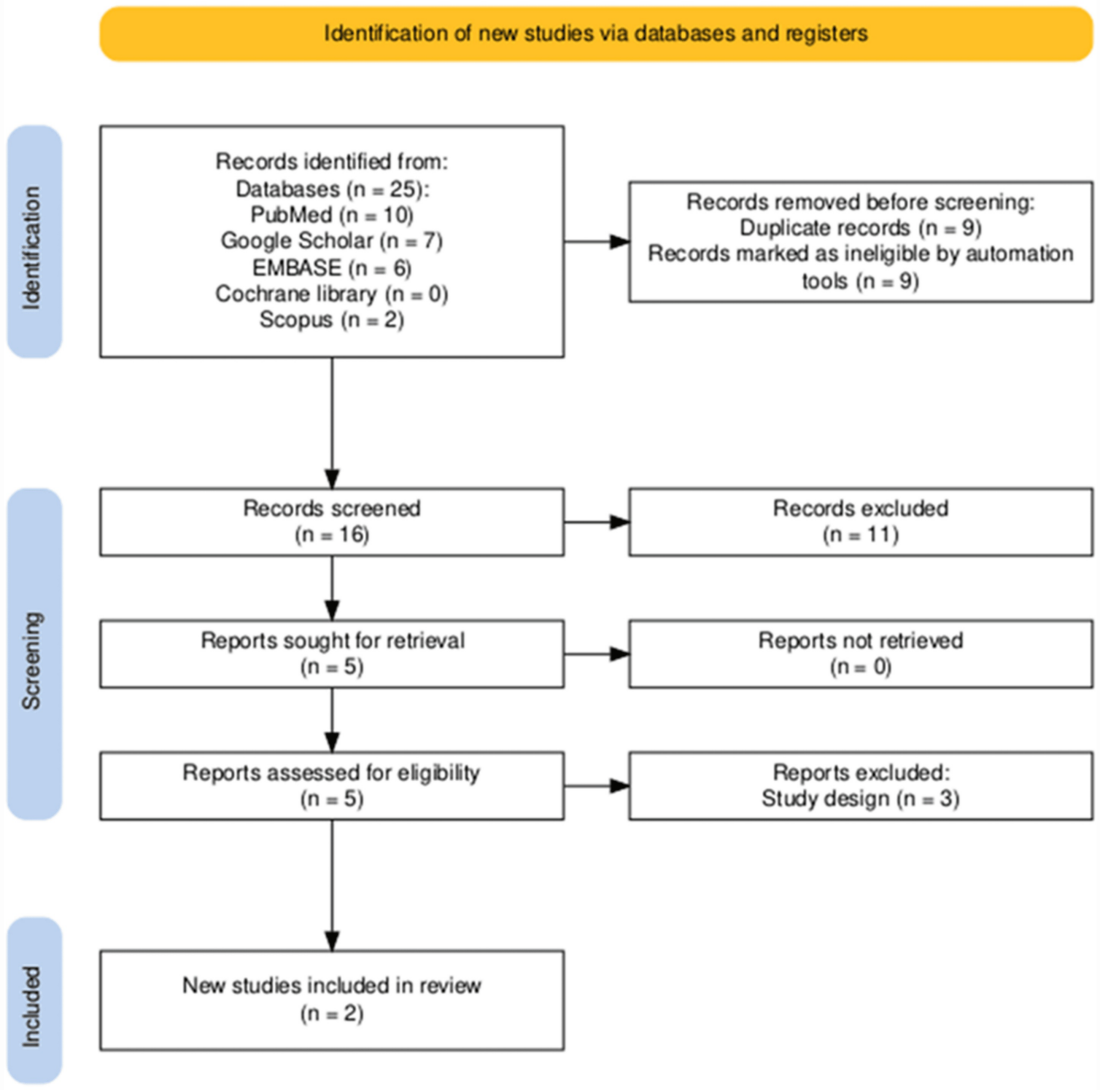
PRISMA protocol for article selection PRISMA, Preferred Reporting Items for Systematic Reviews and Meta-Analyses

Our search terms across the various databases identified 25 articles, which underwent subsequent title and abstract screening. Nine of these were duplicates and were removed. Five articles were then found to be eligible for a full-text review. Three out of five were excluded for the wrong study design. The remaining two studies were eligible for the review. This highlights a significant paucity of data on this subject over a span of over two decades (2000-2024). Figure 1 shows the article selection process according to the PRISMA protocol.

The eligible studies included 264 patients across two retrospective studies. They were published in 2004 and 2020 and looked into patients who had testicular cancer and underwent subsequent subinguinal orchidectomy. One of the studies was a comparison with a cohort of patients who underwent inguinal orchidectomy. Both studies were published in English. The characteristics of the studies and outcomes of interest are summarized in Table 1. 

**Table 1:** Characteristics of included articles

No	Study	Study design	Sample size	Subinguinal orchidectomies		Inguinal orchidectomies	Unsatisfactory control with subinguinal orchidectomy	Unsatisfactory control with inguinal orchidectomy	Complications in low cord	Key findings
1	Anderson et al., 2020 [7]	Retrospective observational	42	42		0	4	0	4	Three patients had involvement of the spermatic cord, and one patient had a positive surgical margin. Four patients developed postoperative seromas, of which three required drainage. No patients experienced neuropathic complications, hernias, or wound breakdown
2	Ashdown et al., 2004 [5]	Retrospective cohort study	222	Stage 1 tumor	85	73	11	9	0	Analysis showed no statistically significant difference in relapse or mortality rates between high and low-cord orchidectomy for clinically stage 1 tumors. For stage 2-4 tumors, the sample size was insufficient to assess the statistical significance of differences in relapse or survival. No significant complications were observed with the low-cord orchidectomy approach.
		-		Stage 2-4	17	47	3	14		

Oncological Outcomes 

Of the 264 patients, 54.75% (n=144) underwent a subinguinal or low-cord orchidectomy. Among them, 12.5% (n=18) experienced unsatisfactory oncological outcomes during follow-up. These outcomes included cancer relapse (78%, n=14), spermatic cord invasion (SCI) (16.5%, n=3), and a positive resection margin (5.5%, n=1). As the included studies also had a high-cord orchidectomy arm (n=120), the outcomes of these patients were reviewed as well. Among this cohort, 19.1% (n=23) had unsatisfactory oncological control during follow-up, in the form of cancer relapse. Of note, in the case-control series, high-cord orchidectomy was performed in patients with stage 2-4 tumors. A Fisher’s exact test was performed to compare recurrence rates between subinguinal and inguinal orchidectomies in both stage 1 and stage 2-4 tumors, based on data reported by Ashdown et al. [5] The test yielded a p-value of 1.0 for stage I tumors and 0.52 for stage 2-4 tumors, indicating no statistically significant difference in oncological outcomes between the two surgical approaches across both stages. Both studies had a mean follow-up period of approximately 40 months.

Anderson et al. [7] did not compare low-cord orchidectomies to high-cord procedures, and Ashdown et al. [5] found no significant differences in oncological control between the two surgical approaches.

Complication Rates

The study by Anderson et al. [7], which examined 42 subinguinal orchidectomies, reported a 9.5% complication rate (n=4), all due to seroma formation. Three of these patients required surgical drainage. Ashdown et al. [5] did not report on complication rates.

Risk of bias assessment

Risk of bias assessment was done using the Newcastle-Ottawa Scale (NOS) [13]. The study by Ashdown et al. had a low risk of bias, as it scored higher across multiple domains due to a clearer cohort design and longer follow-up duration. Anderson et al., on the other hand, scored lower on the NOS due to the absence of a comparator or control group, limited cohort design, and shorter follow-up periods (Table 2).

**Table 2:** Risk of bias assessment using the Newcastle-Ottawa Scale ^✘^Did not meet the criterion ^★^Met the criterion ^★★^Met the criterion to a high level

Criteria	Ashdown et al., 2004 [5]	Anderson et al., 2020 [7]
Selection		
Representativeness of the exposed cohort	★	★
Selection of the non-exposed cohort	★	✘
Ascertainment of exposure	★	★
Outcome of interest not present at start	★	★
Comparability		
Comparability of cohorts	★★	✘
Outcome		
Assessment of outcome	★	★
Follow-up long enough for outcomes	★	✘
Adequacy of follow-up	★	★
Total score	9/9	5/9
Risk of bias	Low	Moderate

Discussion

The role of inguinal orchidectomy in managing suspected testicular malignancy has long been established as the standard of care [7]. Testicular lymphatic drainage is directed to the para-aortic nodes, unlike the scrotum, which drains to the superficial inguinal lymph nodes. The difference in drainage routes is the basis on which an inguinal orchidectomy is preferred over a scrotal orchidectomy [5]. While the conventional high-cord inguinal orchidectomy is a straightforward and generally well-tolerated procedure, the subinguinal or low-cord orchidectomy is a less invasive approach with fewer proposed risks of complications [5,7,14,15].

The scrotum's nerve supply is complex, with sensory innervation primarily from the ilioinguinal nerve (anterior scrotum), the genital branch of the genitofemoral nerve (anterior and lateral scrotum), and the posterior scrotal branch of the pudendal nerve (posterior scrotum). Autonomic innervation is provided by sympathetic fibers from the hypogastric plexus, which regulate smooth muscle tone and blood flow [16,17]. 

Chronic scrotal pain can be a debilitating condition that follows scrotal surgeries, including varicocelectomies, hernia repairs, or orchidectomies [18]. Post-surgical pain often arises from nerve injury, entrapment, or fibrosis, leading to hyperalgesia or allodynia in the affected region [19]. Inflammation and scar tissue in the area can also compress the nerves, contributing to chronic pain [20]. Treatment may include nerve blocks, analgesics, and, in refractory cases, surgical revision or neurolysis [21].

High-Cord Approach Versus Low-Cord Approach

In the high-cord approach, the external oblique aponeurosis is divided to expose the contents of the inguinal canal and locate the deep inguinal ring. The affected testicle is excised along with the spermatic cord, which is ligated at the level of the deep inguinal ring [7]. This approach, while providing comprehensive excision of malignancy, risks damaging the fine nerves within the inguinal canal, namely the ilioinguinal nerve and the genital branch of the genitofemoral nerve, which can lead to complications as previously mentioned. Further complicating the matter, the ilioinguinal nerve demonstrates considerable anatomical variability, making its course unpredictable [22].

The subinguinal (low-cord) approach is a modification of the conventional high-cord inguinal orchidectomy. In this technique, the affected testicle and part of the spermatic cord are excised at the level of the superficial inguinal ring [5]. This method avoids the need to incise the external oblique aponeurosis and open the inguinal canal, thus reducing the risk of nerve injury. Randomized studies by Gontero et al. and Al-Kandari et al. have demonstrated that the subinguinal approach for varicocelectomy results in less postoperative pain and lower complication rates compared with the inguinal approach [9,10]. While it is important to acknowledge that varicoceles and testicular cancer are two different pathologies, these findings may hold potential for testicular cancer, where up to 25% of survivors experience phantom testis pain [23]. Sparing the external oblique aponeurosis also makes the subinguinal approach a less invasive technique that is quicker and easier to perform, with a potentially shorter postoperative recovery period.

How Common Is Spermatic Cord Invasion?

An inguinal approach with high ligation of the spermatic cord at the deep inguinal ring might better contain the lymphatic and local spread of malignancy. However, this has not been conclusively demonstrated, with the management of testicular cancer increasingly relying on radiographic and serological markers rather than surgical margins alone [5,24,25]. Haroon et al. retrospectively analyzed the histopathology of 121 patients who underwent inguinal orchidectomy and found that only three (4%) had proximal SCI, which would have necessitated high-cord ligation [14]. The authors proposed that the subinguinal approach could serve as a less invasive alternative for clinically stage 1 tumors.

Sarikaya et al. [15] conducted a similar study involving 200 patients and found that only six patients (3.1%) had proximal SCI, which would have necessitated a high-cord approach. They concluded that, due to the low probability of SCI, subinguinal orchidectomy could safely reduce morbidity for stage 1 tumors. These findings align with the results reported by Ashdown et al. [5], included in our review, which showed no significant differences in oncological outcomes between high- and low-cord approaches for stage 1 tumors. A recent histopathological study by Kilinc et al. [26] that looked at 121 testicular tumors found that SCI was rare, occurring only in 4% of specimens (n=5).

Role of Chemotherapy

SCI has historically been considered a key factor in staging and prognosis for testicular cancer, often signifying more advanced disease and a greater risk of occult metastasis [27]. This has traditionally warranted more aggressive treatment strategies. However, advances in testicular cancer management, particularly the widespread use of platinum-based chemotherapy such as BEP (bleomycin, etoposide, and cisplatin), have significantly reduced the clinical impact of SCI [28,29].

These chemotherapy regimens are highly effective at targeting micrometastatic disease, which is often the main concern in patients with SCI, thereby reducing recurrence risk. Studies show that adjuvant chemotherapy after orchidectomy substantially lowers relapse rates, even in patients with high-risk features. For instance, Cullen et al. [29] reported that a single cycle of BEP in high-risk stage I non-seminomatous germ cell tumors (NSGCT) resulted in a two-year malignant recurrence rate of just 1.3% (95% CI: 0.3-3.7%). Similarly, Behnia et al. [30] found that two cycles of BEP in pathological stage II NSGCT, including patients with SCI, led to only one relapse among 82 evaluable patients.

The overall survival benefit of platinum-based chemotherapy has been profound, with five-year survival rates now exceeding 95% [3,6,31]. As a result, while SCI remains a marker of more advanced disease, its prognostic weight has lessened. Effective systemic treatments now allow for curative management of early metastatic spread, reducing reliance on extensive surgical intervention. This shift has reshaped the clinical approach to testicular cancer, making aggressive resection less crucial in initial management. It reinforces the importance of balancing oncological control with efforts to minimize surgical morbidity, particularly in younger patients, where preserving quality of life is paramount.

Patient-Centered Outcomes

Beyond oncological outcomes, increasing attention should be paid to patient-reported outcomes such as postoperative pain, return to normal activity, and sexual function. These factors are particularly relevant in testicular cancer, which predominantly affects young men under the age of 40 [2,3]. Berkowitz et al. found that higher postoperative pain levels are strongly associated with lower patient satisfaction across various surgical procedures, indicating that effective pain management is essential for improving patient experiences [32]. This is consistent with findings from Huang et al., who reported that effective pain management in scrotal surgeries, such as microsurgical varicocelectomy, leads to high satisfaction rates [33]. Studies have shown that patients undergoing less invasive procedures, such as subinguinal varicocelectomy or nerve-sparing techniques, report higher satisfaction rates post-surgery [9,10]. Extrapolating this to the context of orchidectomy, a less invasive approach like subinguinal orchidectomy may offer similar advantages in terms of early return to work, reduced psychological distress, and improved quality of life outcomes, which deserve further exploration in future trials.

Limitations and recommendations

Our systematic review has several limitations that must be acknowledged. First, the review includes only two retrospective studies, both of which are inherently subject to selection bias, lack of randomization, and potential confounding factors. These types of studies provide a lower level of evidence compared to prospective trials or randomized controlled trials (RCTs). Moreover, on the NOS, one of the studies was assessed to have a moderate risk of bias. Although our review provides useful insights, the limited number and retrospective design of the studies reduce the generalizability and strength of the conclusions drawn.

Based on our findings, we recommend that subinguinal orchidectomy be considered in selected patients with early-stage testicular tumors, particularly those with no radiographic or clinical suspicion of proximal spermatic cord involvement. The approach offers several potential benefits, including ease of access, reduced operative time, less postoperative pain, and a lower risk of nerve injury. These factors make it an attractive alternative in specific clinical contexts. However, until stronger evidence becomes available, such recommendations must be cautiously interpreted and tailored to individual patient scenarios.

## Conclusions

Currently, available evidence does not demonstrate a clear difference in oncological outcomes between high-cord and low-cord orchidectomy, although the data remains limited. Some postoperative histopathological studies have suggested comparable findings; however, the overall evidence base is insufficient to draw definitive conclusions about the safety or efficacy of the subinguinal approach. Further investigation, ideally through prospective trials or large-scale retrospective studies, is needed to better define the role of low-cord orchidectomy in the management of testicular cancer.

There remains an unmet need for clarity in determining which surgical approach offers the best balance between oncological safety and patient comfort. Future research should focus on the development and execution of well-designed RCTs comparing subinguinal and inguinal orchidectomy approaches. These studies should include robust oncological and functional outcomes, including patient-reported pain and quality-of-life metrics. If future evidence confirms equivalent oncologic control with reduced morbidity, the subinguinal approach could lead to a paradigm shift in the surgical management of early-stage testicular cancer. Subinguinal orchidectomy may represent a step forward in refining surgical care for testicular cancer patients, especially those who prioritize quicker recovery and fewer postoperative complications. At present, caution and clinical judgment remain essential, but with time and further research, this technique may become an established part of standard urological practice.
